# Polysaccharides with Arabinose: Key Players in Reducing Chronic Inflammation and Enhancing Immune Health in Aging

**DOI:** 10.3390/molecules30051178

**Published:** 2025-03-06

**Authors:** Patricia Pantoja Newman, Brenda Landvoigt Schmitt, Rafael Moura Maurmann, Brandt D. Pence

**Affiliations:** College of Health Sciences, University of Memphis, Memphis, TN 38152, USA; blndvgts@memphis.edu (B.L.S.); rmmrmann@memphis.edu (R.M.M.)

**Keywords:** inflammaging, polysaccharide, arabinose, innate immune system

## Abstract

Aging is associated with a decline in physiological performance leading to increased inflammation and impaired immune function. Polysaccharides (PLs) found in plants, fruits, and fungi are emerging as potential targets for therapeutic intervention, but little is known about their effects on chronic inflammation and aging. This review aims to highlight the current advances related to the use of PLs, with the presence of arabinose, to attenuate oxidative stress and chronic and acute inflammation, and their immunomodulatory effects associated with antioxidant status in monocytes, macrophages, and neutrophil infiltration, and leukocyte rolling adhesion in neutrophils. In addition, recent studies have shown the importance of investigating the ‘major’ monosaccharide, such as arabinose, present in several of these polysaccharides, and with described effects on gut microbiome, glucose, inflammation, allergy, cancer cell proliferation, neuromodulation, and metabolic stress. Perspectives and opportunities for further investigation are provided. By promoting a balanced immune response and reducing inflammation, PLs with arabinose or even arabinose per se may alleviate the immune dysregulation and inflammation seen in the elderly, therefore providing a promising strategy to mitigate a variety of diseases.

## 1. Introduction

Aging represents the major risk factor for infections and chronic disorders, including cancer, cardiovascular, neurodegenerative, and autoimmune diseases [1]. This biological process is characterized by the progressive decline in overall physiological homeostasis and performance, ultimately leading to death. Age-related changes in the immune system, termed as immunosenescence, have been implicated as central players in these processes, as chronic low-grade inflammation represents a common feature on aged tissues and age-related disorders [2,3]. In fact, this phenomenon, known as inflammaging, has been pointed out as a driver of metabolic diseases, autoimmunity, and frailty in the elderly, and has been correlated with increased morbidity and mortality [4,5]. The driver of inflammaging is mostly attributed to the dysregulation of adaptative immunity and the compensatory overactivation of innate immune responses combined with tissue damage accumulation [6]. In this regard, targeting these imbalanced mechanisms may be a promising strategy to control inflammation in aging.

In recent years, there has been a surge of research focused on polysaccharides (PLs) and their impact on chronic inflammation, which reflects an increasing awareness of their potential applications as therapeutic interventions. These bioactive molecules are found in plants, fungi, and algae and have been demonstrated to exert protective effects in inflammaging, as exemplified by recent studies with *Radix astragali* [7], *Cannabis sativa* [8], ginseng extract [9], and *Ximenia americana* [10]. More specifically, PLs have been associated with direct effects on immune function, as demonstrated by treatments with American ginseng root [11], Isatis root [12], *Ganoderma* sp. [13], and *Astragalus* sp. extracts [14]. In addition, PLs have been clinically tested for their excellent biocompatibility, structural stability, and biodegradability [15].

Despite current advances, the full elucidation of PLs’ biological properties, targets, and tissue interactions is still required [15]. In this regard, we will focus on examining the recent advances in PL treatments involving inflammaging and immune function. In addition, we intend to highlight the importance of arabinose, a monosaccharide, which is present in fungi [16], fruits [17], and plant extracts [18], and to delimit the current understanding of its therapeutic potential in monocytes and neutrophils.

## 2. Inflammaging and Innate Immune System

As described above, inflammaging is a state of chronically elevated inflammatory mediators observed in the serum of older adults, which differs from infection-related acute inflammatory states in that it is low-grade and sterile [3]. Increased levels of IL-1β, IL-6, TNFα, and C-reactive protein (CRP) are often described as markers of inflammaging [19], which can be traced to myeloid cells such as monocytes and macrophages [20]. Indeed, these innate immune cells have been speculated to be one of the major sources of pro-inflammatory cytokines in aged tissues [20,21]. The dysregulation of this immune compartment is mostly associated with the chronic, repetitive, lifelong antigenic stimulation by exogenous (e.g., pathogens) and endogenous (e.g., neoplastic cells and cellular debris) inflammatory stimuli, ultimately culminating in altered immune responses in older age [19]. In parallel, aged tissues accumulate senescent cells, which fuel the dysregulated inflammatory responses of innate immune cells through their constant secretion of cytokines and chemokines plus associated molecular damage mediators [22]. While other mechanisms have been implicated in the inflammaging development (e.g., gut leakage) [22], we aim here to summarize the age-related adaptations in the innate compartment that contribute to this phenomenon.

Monocytes and macrophages are key players involved in the production of inflammatory mediators in multiple contexts [21,23]. Aging in the monocyte population is described by an increased proportion of intermediate (CD14^low^CD16^+^) and non-classical (CD14^−^CD16^high^) monocytes, characterized by a higher production of IL-1β, IL-6, and TNFα under basal conditions and up-stimulation [24,25]. Furthermore, monocytes isolated from aged individuals exhibit dysregulation of TLR-associated responses [24,26,27]. While TNFα and IL-6 are reduced upon TLR1/2 stimulation, the opposite is observed regarding TLR4 stimulation [26]. On the other hand, IL-8 production is higher after the stimulation of TLR1/2, TLR2/6, TLR4, or TLR5 [27]. These variable responses have been correlated with the altered downstream activation of MAPK and ERK1/2 pathways [26,27]. In addition, recent studies have also evidenced an association between defects in cellular metabolic processes and changes in immune function, as demonstrated by the co-occurrence of mitochondrial respiration impairment and abrogated monocyte LPS response in older adults [28,29].

Similarly, macrophages isolated from aged tissues exhibit metabolic alterations related to impaired immune function. For instance, NAD+ production declines in these cells, a molecule required for sirtuin activity, thus correlated with diminished SIRT2-dependent inflammasome inhibition and the increased activation of this pathway in aging [30,31]. In addition, the accumulation of dysfunctional mitochondria on aged macrophages has been directly correlated with the increased basal stimulation of the NLRP3 inflammasome and cGAS-STING pathways, driven by the cytoplasmic leakage of mitochondrial DNA and reactive oxygen species (ROS) [32,33,34]. Autophagy defects also contribute to enhancing endoplasmic reticulum (ER) stress and mitochondrial dysfunction, further leading to NLRP3 activation and increased expressions of IL-1β and IL-18 [35]. In fact, the inhibition of NLRP3 attenuates several age-related changes in macrophages that contribute to inflammaging [36]. Finally, it has recently been observed that aged macrophages exhibit increased cyclooxygenase 2 (COX2) expression and prostaglandin 2 (PGE2) production, which correlates with an increased expression of inflammatory cytokines such as TNFα and IL-6 [37].

Another key innate immune cell compartment is neutrophils, the first responders to infection. Neutrophils are components of the white blood cells responsible for defending our bodies against infection, constituting 60% to 70% of all leukocytes in human blood. Neutrophil recruitment is mediated by the release of damage-associated molecular patterns (DAMPs) from damaged cells or pathogen-associated molecular patterns (PAMPs) during infection [38]. Their recruitment contributes to tissue repairment, the phagocytosis of necrotic tissue, and the control of the recruitment of additional neutrophils. Monocytes/macrophages interact with neutrophils by releasing chemoattractants that recruit neutrophils to the site of inflammation. In a reverse mechanism, once neutrophils reach the site of inflammation, they recruit more monocytes and induce their differentiation into macrophages, increasing inflammation [39]. Neutrophils form neutrophil extracellular traps (NETs) as a defense mechanism, and NETosis leads to neutrophil death, exposing all neutrophil contents and increasing inflammation [40].

Aging is primarily associated with a defective migratory capacity in this population, which is attributed to the constitutive activation of the lipid kinase phosphoinositide 3-kinase (PI3K) [41]. This phenotype has been implicated in both reduced wound healing and in situ infection control due to decreased neutrophil recruitment [42]. In addition, diminished tissue egress is observed, which correlates with increased local inflammation, as demonstrated in burn-associated lung injury in aged mice [43]. Apart from defective migration, aged neutrophils display increased basal ROS, cytokine, and metalloproteinase production, associated with persistent nuclear factor kappa B (NF-kB) activation [44,45]. An increased proportion of these basally activated cells is further facilitated by impaired macrophage efferocytosis, which is required to remove senescent neutrophils from the circulation [46].

## 3. Polysaccharide Extraction, Separation, and Purification

As is known, PLs are distributed in plants, fungi, and algae. The extraction methods, separation, purification, and characterization potentiate the natural effect and increase the chances of developing new pharmacological targets. Huang and colleagues developed a guideline where common extraction methods using hot water, acid, or alkaline aqueous solution are the most useful extraction methods for PLs [47]. The PLs present in this review, as described in Table 1, are predominantly extracted using water, hot or cold. PLs such as *Ximenia americana* added steps for deproteinization intending to keep PLs less contaminated. Methods such as the DEAE-cellulose column, high-performance liquid chromatography (HPLC), and column chromatography are examples of techniques used to purify the PLs and even prepare fractions of the major monosaccharides present in a complex PL structure [47]. Table 1 shows the methods of extraction, separation, and purification used by the PLs discussed in this review, and the majority of PLs show the presence of arabinose in their structure.

## 4. Polysaccharides and Inflammaging

PLs have been recently demonstrated to exert distinct effects on the aging process. These complex carbohydrates found in plants, fungi, and algae have shown potential as anti-aging agents due to their antioxidant, immunomodulatory, and anti-inflammatory properties [71,72,73]. PLs, such as β-glucans and fucoidans, have been increasingly studied for their ability to delay and/or reverse age-related physiological decline [74]. PLs from *Cardyceps cicadae* [55], ginseng extract [9], *Astragalus membranaceus*, and *Ganoderma lucidum* are able to scavenge ROS and reduce oxidative damage, a major driver of aging [75]. Additionally, PLs possess anti-inflammatory properties, as exemplified by PLs extracts from *Ximenia americana* reducing IL-8 and leukocytes migration [18,76]. PLs from medicinal mushrooms and seaweeds have also demonstrated the ability to modulate immune function by enhancing the activity of macrophages and natural killer cells [77,78]. In summary, by promoting a balanced immune response, PLs may mitigate the immune dysregulation seen in the elderly.

On the other hand, PLs can act as immunomodulators by increasing anti-inflammatory cytokines and decreasing pro-inflammatory cytokines. For instance, β-glucans from oats (*Avena sativa*) and mushrooms (*G. lucidum*, *Lentinula edodes*) have been shown to suppress the release of IL-6 and TNFα while promoting the production of anti-inflammatory cytokines such as IL-10 [79]. In addition to their anti-inflammatory properties, PLs from larch trees, *Fucus vesiculosus*, *Undaria pinnatifida*, *Chondrus crispus* [80], *G. lucidum*, *Flammulina velutipes*, and *Sparassis crispa* [81] and *Cucurbita moschata* [82], *Amorphophallus konjac* [70], and kappa-carrageenan [53] also support gut health by acting as prebiotics and promoting the growth of beneficial gut microbiota. This not only improves immune function but also reduces systemic inflammation [81], providing a multifaceted approach to managing inflammaging and extending health span.

Modulating immune function is another important role of PLs, orchestrated by interactions with specific pattern recognition receptors (PRRs) and TLR on immune cells [83,84]. PLs, as isolated from *G. lucidum*, enhanced immune activity by improving phagocytosis, regulating cytokine production and decreasing pro-inflammatory cytokines such as IL-6 and TNFα while promoting anti-inflammatory cytokines like IL-10 [85]. Wenfung Li and colleagues showed *Astragalus* sp. polysaccharide (APS) to exert anti-tumor activity by improving the phagocytosis of peritoneal macrophages and regulating cytokine production, denoted by decreased pro-inflammatory cytokines such as IL-2, TNFα, and interferon-γ (IFN-γ) [86]. This effect helps to protect tissues from damage and prevents chronic inflammation associated with age-related diseases, positioning PLs as potential therapeutic agents for managing inflammatory conditions while enhancing immune resilience and counteracting inflammaging.

From all the PLs presented in Table 2, 87% presented arabinose in their structures. Only 13% did not show the presence of arabinose, such as *Amorphophallus konjac* [70], *Aparassis crispa* [62], and *Carrageenan* [53]. Considering the PLs with arabinose in their structure, 60% show arabinose as the main or second main compound in their composition such as *Ximenia americana* [18], *Saposhnikoviae radix* [40], *Ganoderma lucidum* [48], and *Fragaria* x *ananassa* [67]. We will focus on PLs with the presence of arabinose on their structure, evaluating the interaction between monocytes and neutrophils and possible mechanisms involving arabinose as a pharmacological target.

## 5. Polysaccharide Interaction with Monocytes and Neutrophils

PLs from various natural sources, such as mushrooms, algae, and medicinal plants, have shown potential in modulating the activity of aged monocytes [85,104]. PLs, particularly from *A. sative*, *Hordeum vulgare*, and Reishi and Shiitake mushrooms, have been reported to reduce the production of pro-inflammatory cytokines and enhance the anti-inflammatory response of monocytes in aged individuals [58]. This immunomodulatory effect is linked to the activation of PRRs such as Dectin-1 on monocytes, which triggers an anti-inflammatory signaling pathway [83]. Studies with *Fragaria* x *ananassa*, *Morus alba*, and *G. lucidum* have shown that polysaccharides can reduce oxidative stress and promote the secretion of anti-inflammatory cytokines such as IL-10 in aged monocytes, thereby counteracting inflammaging and improving immune function [97,105].

As discussed above, one of the major challenges of aging is the declining ability of monocytes to respond appropriately to pathogens and tissue damage. PLs from *Dendrobium* spp. have been studied as potential agents for rejuvenating aged monocytes by enhancing their phagocytic activity and modulating cytokine secretion [99]. Similarly, PLs from mushrooms have been shown to modulate cytokine secretion at a level that initiates signaling cascades critical for immune responses [106]. For instance, fucoidan, a PL found in brown algae, has been shown to improve aged monocyte function by reducing the expression of pro-inflammatory genes and increasing anti-inflammatory signaling pathways [60,89]. Furthermore, PLs modulated the NF-κB signaling pathway, reducing excessive inflammation and ensuring a controlled immune response, especially in chronic inflammatory conditions [81]. These findings suggest that polysaccharides may help restore the immune balance in aging, promoting healthier aging and improved immune responses.

Given their ability to modulate aged monocytes and reduce inflammaging as described before, PLs have considerable potential as therapeutic agents for age-related immune dysfunction. In addition to acting as immunomodulators, PLs have antioxidant and tissue-regenerative properties that may further support the function of aging monocytes [70]. Clinical studies are exploring the use of PLs to reduce chronic inflammation in elderly patients, particularly in conditions such as cardiovascular disease, arthritis, and neurodegenerative disorders in which monocyte-driven inflammation plays a key role [86]. By targeting the underlying causes of monocyte aging and dysfunction, PLs may represent a novel class of immunomodulatory drugs aimed at promoting healthy aging and reducing the burden of age-related disease.

Over the past 20 years, several papers have described the mechanisms by which PLs coordinate neutrophil phenotype and function. Ming-Jen Hsu and colleagues showed that PLs from *G. lucidum* (PS-G) enhanced phagocytic activity through multiple signaling pathways, such as Protein kinase C (PKC), Src family, and p38-MAPK, and induced neutrophil migration in isolated cells and differentiated HL-60 [48]. Meanwhile, PLs from *Rosa davurica* (RDPA1) inhibited human neutrophil migration in vitro and impaired neutrophil infiltration in mouse peritonitis [49]. Similarly, PLs from *Ximenia americana* bark (TPL-Xa) reduced inflammation by reducing leukocyte rolling, adhesion, cytokine IL-8, and increased IL-4 in a murine model of gastritis [18].

PLs present in *Astragalus* sp. extract powder, commonly commercialized as bulk supplement, were demonstrated to control the neutrophil-to-lymphocyte ratio (NLR), a prognostic marker in patients with cancer receiving immunotherapy, decreasing NLR in 31.60% upon intravenous PG2, *Astragalus* sp. root PLs, and normalizing the NLR in patients with lung cancer in combination with immunotherapy [50]. Jie Zhang and colleagues tested PLs from *Hedyotis diffusa* (HDP) in acute lung injury induced by LPS and demonstrated attenuated in vivo inflammatory parameters, characterized by reduced IL-6 and TNFα levels, leukocyte counts, and NET formation [52]. Recently, root-derived PLs from *Saposhnikoviae radix* (SP40015A01) have been shown to improve neutrophil density and reduce inflammatory factor levels [40].

## 6. Arabinose: A Promising Anti-Inflammatory and Immunomodulatory Agent

The evidence presented above on how PLs, with the presence of arabinose, ameliorate inflammaging and modulate immune function brings us to the question of how the arabinose present in the PL itself could be involved in the healing process. Polysaccharides are chains of small molecules called monosaccharides. The most nutritionally important monosaccharides are those with pentoses and hexoses, i.e., molecules with five and six carbons in the backbone [107]. In the last 10 years, we have seen an increase in research on the effects of monosaccharides found in fruits, vegetables, and algae that can help prevent premature aging and its complications. The combination of monosaccharides such as D-arabinose and galactose inhibits peripheral inflammatory nociception in a mouse model [108], arabinoxylan, a combination of arabinose and xylose, reduced LPS-induced inflammation, and showed an immunomodulatory effect in RAW264.7 macrophage cells [109], and beet pulp rich in D-galacturonic acid and L-arabinose shows potential applications in diabetes, blood sugar regulation, and antimicrobials [110].

It is important to note that monosaccharides can form optically active stereoisomers, meaning that the direction of light determines whether the structure rotates to the right (D) or left (L), thus defining D or L stereoisomers [107]. It is a water-soluble aldose with an aldehyde group (-CHO) at the end of the molecule. A reducing reaction can bring the molecule to a primary alcohol, and under certain conditions, it can be isomerized to other sugars such as ribose or xylose [111,112].

**Figure 1:**
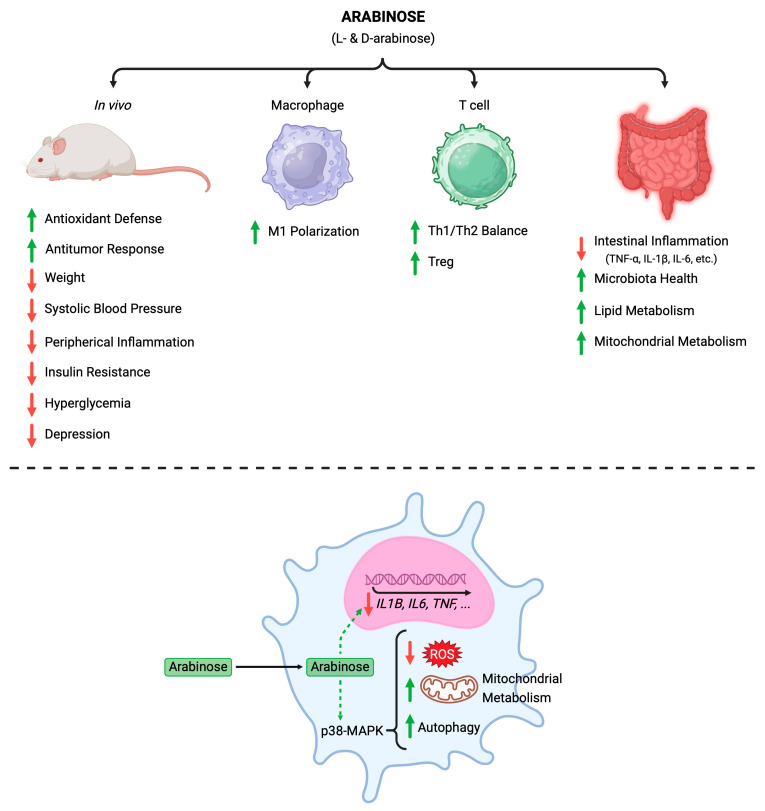
Effect of arabinose in different models.

Arabinose forms both D- and L-arabinose and has been studied for its pharmacological effects in various disease models such as metabolic syndrome [113,114,115], inflammation [116,117], food allergy [118], antioxidant [119], cancer [120], antidepression [121], and metabolic stress [122], as shown in Figure 1.

A review published by Csaba Fehér presented L-arabinose as a blood-sugar-reducing agent, an antioxidative agent with protective activities against hyperglycemia, and a precursor for antiviral drug development [123]. L-arabinose was administered for 6 weeks, and rats with high fat diet-induced metabolic syndrome had a marked reduction in body weight, systolic blood pressure, diastolic blood pressure, fasting blood glucose, triglycerides, total cholesterol, serum insulin, TNFα, and leptin [113]. Furthermore, L-arabinose also modulated gene-expression related to lipid metabolism and mitochondrial function in key metabolic tissues through the regulation of mice microbiota. It has also been shown to inhibit colitis through the microbiome modulation [114,115].

To understand the effect of arabinose on the immune system, Luyuan Kang and colleagues supplemented mice diets with L-arabinose in an LPS inflammatory model to evaluate intestinal inflammation [117]. They observed that L-arabinose downregulated serum TNFα, IL-1β, and IL-6 as well as gene expression levels of TNFα, IL-1β, IFN-γ, and TLR4 in the jejunum and colon and increased the concentration of short-chain fatty acids. Another study using L-arabinose pretreatment to attenuate gliadin-induced food allergy in mice showed that it improved the Th1/Th2 immune response imbalance based on the expression levels of key related cytokines and transcription factors in the small intestine and spleen of sensitized mice via regulation of tight junction proteins (TJ) and the suppression of p38-MAPK and p65/NF-κB inflammatory signaling pathways [118].

Recently, a switch between L- and D-arabinose has also been reported. Recent publications have shown the effect of D-arabinose on in vitro and in vivo mice models [118,120]. A publication by Tang and colleagues using an in vitro model of breast cancer showed that 50 mM of D-arabinose induced cytotoxicity modulated by autophagy and the p38-MAPK pathway [120]. Similarly, a mechanism of action of L-arabinose through the p38-MAPK regulation of T cells was reported by Wang et al. [118]. In tests with SH-SY5Y human neuroblastoma cells and the BV2 mouse microglial cell line in conjunction with behavioral tests, D-arabinose induced CREB regulated transcription coactivator 1 (CRTC1) expression through the expression of peroxisome proliferator-activated receptor gamma (PPARγ) and transcription factor EB (TFEB) and also enhanced Acyl-coenzyme A synthetase short-chain family member 2 (ACSS2)-dependent CRTC1 transcription by activating AMP-activated protein kinase (AMPK) through the lysosomal AXIN-liver kinase B1 (LKB1) pathway [121].

Arabinose can be reduced to a sugar alcohol called arabitol [111] and has been shown to have anti-inflammatory effects. The presence of D-arabitol on the alkaloids SZ-A showed anti-inflammatory effects in macrophage cell line RAW 264.7 in an LPS-induced model [124], Li and colleagues showed that the effect of D-arabitol has ameliorated body weight fat gain, fat accumulation, insulin resistance, and gut microflora, indirectly promoting AMPK-PGC-1a-related white adipose tissue [125].

## 7. Future Perspectives

The papers summarized in this review are among the first to demonstrate that polysaccharides found in fungi [16], fruits [17], and plants [18] have therapeutic potential in monocytes and neutrophils through anti-aging, antioxidant, anti-inflammatory, and immunomodulatory activities [8,9,11,13,14]. However, there is still a promising opportunity to advance research. As mentioned above, the mechanisms of how the polysaccharides act in in vivo or in vitro are still unknown. Also, the monosaccharides, small molecules that make up the polysaccharide chain, have tremendous importance in their biological activity [117,119,120,121].

Additionally, as the evidence presented above, PLs are able to reduce pro-inflammatory cytokines secretion and expression [60,89,97], exert immunomodulatory effects [83], and enhance phagocytosis [99] and antioxidant status in monocytes and macrophages, key players involved in the production of inflammatory mediators in multiple contexts [21,23]. PLs have been shown to reduce key factors responsible for dysfunction in aged monocytes [58,97,105], although there are still inconclusive links between aging, chronic inflammation, and monocyte phenotypes that should be further investigated. PLs also showed effects on neutrophils, another key innate immune cell, such as reduced neutrophil migration through the p38-MAPK pathway [49], neutrophil infiltration [48], leucocyte rolling adhesion [18], an altered neutrophil-to-lymphocyte ratio [49], and reduced inflammatory parameters [50,52]. Notably, the mechanisms of PLs per se in monocytes and neutrophils, key cells involved in inflammaging, have the potential to ameliorate the chronic inflammation present in the elderly population.

The fact that the PLs are composed of monosaccharides raises the question of whether the major monosaccharides present in the PLs chain could act as an anti-inflammatory and immunomodulatory agent in chronic inflammation. Arabinose is a monosaccharide found in PLs derived from, for example, *Ximenia americana* [18], *Ganoderma lucidum* [49,79], hemp residues [8], ginseng [9], and *Astragalus* spp. [86] and has been shown to have pharmacological effects in several disease models including metabolic syndrome [113,114,115], inflammation [116,117], food allergy [118], antioxidant [119], cancer [120], and anti-depression [121].

Authors have published on PLs and pentose sugars, such as arabinose, highlighting the p38-MAPK pathway as a primary mechanism of action [49,118,120]. A reduced sugar alcohol called arabitol also showed indirect effects on the p38-MAPK pathway [124,125]. P38-MAPK directly influences innate immune cells by driving cellular responses related to ROS levels [126]. This perspective opens up opportunities to explore the modulation of monocytes and neutrophils in a series of tests focused on the ROS pathway. Additionally, experiments involving calcium signaling are viable, given that ROS inhibits calcium entry through the ORAI1 channel in monocytes or activates neutrophils through NFAT transcription [127].

## 8. Conclusions

In recent years, studies employing PLs and their major components, the monosaccharides, have shown advances in our knowledge of their impact on chronic inflammation and immunomodulation as presented above. However, when the focus turns to arabinose, a full understanding of the pharmacological effects on the innate immune system and the consequences for inflammaging are still open. Likewise, targeting inflammaging appears to be a promising strategy for treating age-related diseases and disorders. Understanding the mechanisms of PLs with arabinose or even arabinose per se could lead to the development of targeted therapeutics for a variety of age-related diseases.

## Figures and Tables

**Table 1:** Polysaccharide’s extraction methods.

Name	Extraction/Separation	Purification	Ref
*Ximenia americana*	Extracted with NaOH and mass spectrometry (GC-MS).	Ethanol precipitation	[18]
*Saposhnikoviae Radix* (SP40015A01)	Extracted water extraction and DEAE-cellulose. Separation by high performance liquid chromatography (HPLC).	Purified by a Sepharose CL-6B column	[40]
*Ganoderma lucidum* (PS-G)	Extracted with boiling water. Separation by alcohol and gel filtration Sephadex G 50 column.	Purified by anion exchange chromatography with a column of DEAE-cellulose.	[48]
*Rosa davurica* (RDPA1)	Extraction in ethanol and distilled water. Separation by Sepharose CL-6B column (2.6 × 100 cm), eluted with 0.15 M NaCl.	Purified by a Sepharose CL-6B column (2.6 × 100 cm), eluted with 0.15 M NaCl and DEAE-cellulose.	[49]
*Astragalus* (PG-2)	Extracted with hot water. Precipitation by ethanol.	Purified by gel chromatography	[50]
*Astragalus* *membranaceus*	Extracted with cold water. Ethanol precipitation.	Purified by DEAE-cellulose chromatography and a Sephadex G-100 column	[51]
*Hedyotis diffusa* (HD-PS-3)	Extracted with DEAE-52 cellulose column. Separation by NaCl solution.	Purified by an ephacryl S-200 column and distilled water	[52]
*Carrageenan*	Extracted by Qingdao Bozhi Huili Biotechnology Co., Ltd., China. Qingdao Bozhi Huili Biotechnology Co., Ltd., Qingdao, China.	Purified by Qingdao Bozhi Huili Biotechnology Co., Ltd., China	[53]
*Cordyceps cicadae*	Extracted with hot water for 2 h with Sevag reagent. Dialysis.	Purified by DEAE-52 column chromatography	[54,55]
*Panax ginseng*	Extracted with hot water with MAS-II. Ethanol precipitation.	Purified by DEAE-cellulose chromatography	[56]
*Avena sativa*	Extracted with alkaline solution (NaOH) and ultrasound-assisted extraction. Ethanol precipitation.	Purified by gas chromatography	[57,58]
*Lentinula edodes*	Extracted with subcritical water with ultrasound-assisted extraction. Ethanol precipitation.	Purified by gas chromatography	[59]
*Fucus vesiculosus*	Extracted in acid conditions (0.1 M HCl). Ethanol precipitation.	Purified by dialysis	[60]
*Undaria pinnatifida*	Extracted with hot water with Sevag reagent. Ethanol precipitation.	Purified by DEAE-52 column chromatography and Sephacryl S-400 gel	[61]
*Chondrus crispus*	Extracted with soxhlet extraction with diethyl ether. Ethanol precipitation.	Purified by gas chromatography	[62]
*Flammulina velutipes*	Extracted with hot water. Ethanol precipitation.	Purified by DEAE-cellulose chromatography	[63]
*Sparassis crispa*	Extracted in hot water with Sevag reagent. Ethanol precipitation.	Purified by DEAE-52 column chromatography and a Sepharose G-100 column	[64]
*Cucurbita moschata*	Extracted with hot water for 4 h, HCl for 40 min, and NaOH for 10 min. Ethanol precipitation.	Purified by a DEAE-Sepharose Fast Flow column and Toyopearl HW-65F column	[65]
*Hordeum vulgare*	Extracted with enzymatic alkaline extraction. Ethanol precipitation.	Purified by high-performance liquid chromatography	[66]
*Fragaria* x *ananassa*	Hot water with 0.05 M HCl. Ethanol precipitation.	Purified by gel filtration chromatography	[67]
*Morus alba*	Hot buffer, chelating agent, dilute alkali, and concentrated alkali. Ethanol precipitation.	Purified by gas chromatography	[68]
*Dendrobium* spp.	Deionized water. Ethanol precipitation.	Purified by DEAE-cellulose chromatography and a Sephadex G-200 column	[69]
*Amorphophallus konjac*	Hot water with ethanol. Ethanol precipitation.	Purified by dialysis and gel permeation chromatography	[70]

**Table 2:** Polysaccharides properties and anti-inflammatory/immunomodulatory activity.

Name	Source	Molecular Weight (kDa)	Composition	Structure	Methods	Model	Effects	Ref.
* Ximenia *americana*	Bark	Not described	Arabinose, Rhamnose, Galactose, Glucose, Xylose, Manose	Not described	Gastritis induced by indomethacin, histology, biochemical analysis, and intravital microscopy	Gastritis in mouse	Reduced neutrophil migration, pro-inflammatory cytokines, and microscope lesion	[18]
** Saposhnikoviae radix (SP40015A0)	Root (Huacaosheng Traditional Chinese Medicine Co., Hebei, China), named by Professor Zhang Yuan	970	Rhamnose, Galacturonic Acid, Galactose, Arabinose	Composed of 3-α-GalAp, 2-α-GalAp, 2,3-β-GalAp, and 2,3-β-Galp and branched at C3 of 2,3- β-GalAp and C3 of 2,3-β-Galp	Extraction, isolation, purification, structural analysis, neutrophils density, and inflammatory content	Zebrafish	Improved neutrophils density and reduced inflammatory factor content (cytokines and biochemistry analysis)	[40]
* Ganoderma *lucidum* (PS-G)	Mushroom	628–818	Rhamnose Arabinose Xylose Mannose Glucose Galactose	Composed of (1-6)-b-D-glucan, which contains a backbone chain of (1-3)-linked D-glucose residues, five out of 16 D-glucose residues being substituted at O-6 positions with single D-glucosyl units	PS-G purification, neutrophil isolation and HL-60 differentiation, phagocytosis, PKC activity, immunoblotting, immunoprecipitation, and neutrophil transmigration assay	In vitro	Enhance neutrophil function in phagocytosis and chemotaxis	[48]
*Rosa* *davurica* (RDPA1)	Rose Native from Eastern Asia	26.1	Rhamnose, Arabinose, Mannose, Glucose, Galacturonic Acid	Not described	Isolation and purification, neutrophil isolation, transwell, migration and infiltration neutrophil, flow cytometry, and protein binding assays	In vivo in mouse and in vitro	Inhibited in vitro migration of human neutrophils, impacted the migratory behavior of neutrophils, reduced the migrated distance and aver- age velocity of RDPA1-treated cells. Impaired in vivo neutrophil infiltration in the peritonitis mice. Exhibited significant blocking capacity of the inter- action between 2 integrins, and ICAM-1 evaluated and in vitro protein binding assay	[49]
* Astragalus (PG-2)	Root (PhytoHealth Corporation, Taiwan, ROC)	301	Glucose, Arabinose, Fucose, Xylose, Galactose	Composed of α-D-(1,4)-Glc and (1,6)-α-D-Glcp backbone and a branch point at O-6.	Review of patients with lung cancer that used PG-2 as treatment	Lung cancer patients	PG2 could normalize the neutrophil-to-lymphocyte ratio (NLR) in patients with lung cancer receiving immune inhibitor treatment	[50]
* Astragalus *membranaceus*	Herb	12.3	Glucose, Arabinose, Fucose, Xylose, Galactose	Composed of β-(1→4)-linked d-galactopyranosyl residues	Lymphocyte subsets in blood, macrophage pinocytosis, and cytotoxicity assays	Spleen lymphocytes and mouse tumor	Reduces IL-1B and TNFα and enhances T-cell and B-cell activity	[51,75,86]
** Hedyotis diffusa (HD-PS-3)	Herb Chinese medicine	742.2	Mannose, Rhamnose, Glucose, Galactose, Arabinose	Composed of →4,6)-α-Glcp-(1→, →3,4)-α-Glcp-(1→, →4)- α-Glcp-(1→, →4,6)-α-Galp-(1→, →5)-α-Araf-(1→, α-Rhap-(1→, α-Araf-(1→, α-GlcpA-(1→, →4)-β-Manp-(1→, β-Manp-(1→ and →3)-β-Manp-(1→	In vivo LPS-induced lung inflammation, histology, biochemistry, and immunoblotting	Mouse	Attenuated LPS-induced lung injured and inflammatory parameters through inhibition of complement activation	[52]
*Carrageenan*	Red algae	200–800	Rhamnose, Mannose, Glucose, Fucose, Xylose	Composed of (1,3)-linked galactopyranose-4-sulphate and (1,4)-linked 3,6-anhydrogalactopyranose residues	Biochemistry analysis and intestinal microbiota analysis	In vivo mouse	Improved fecundity, showed antioxidant effect, reduced MDA and repressed NF-kB gene, and increased diversity gut microbiota	[53]
*Cordyceps* *cicadae*	Fungi	128	Glucose, Mannose, Arabinose, Galactose	Composed of β-glucans and heteropolysaccharides	Antioxidant activity assays (DPPH, hydroxyl radical, superoxide anion, FRAP) and cell-based studies (ROS, senescence markers), antioxidant enzyme activities, lipid peroxidation, histopathology, and behavioral changes	Macrophage and mouse	Reduced pro-inflammatory cytokines (TNFα, IL-6) and enhanced macrophage phagocytosis and NK cell activity	[54,55,81]
** Panax *ginseng*	Root	207	Glucose, Arabinose, Rhamnose, Galactose	Composed of β-(1→3) and β-(1→6) linkages	Antibacterial activity assay, Western blot, cytokine assays, *Caenorhabditis elegans* using physiological, microbiomics, and transcriptomic approaches	Bacteria, macrophages, *Caenorhabditis elegans*	Inhibited NF-kB and MAPK pathways and enhanced lymphocyte proliferation and cytokine production	[9,56,81]
** Avena sativa	Oats	134–4100	Glucose, Arabinose, Mannose	Composed of linear β-glucans with mixed β-(1→3) and β-(1→4) linkages	ELISA to measure cytokines, flow cytometry to assess cell activation, and 16S rRNA sequencing to investigate gut microbiota	Macrophages and mouse	Reduced TNFα and IL-6, enhanced gut microbiota and immune response, and activated monocytes via dectin-1 receptors	[57,58,79]
*Lentinula edodes*	Mushroom	2.384–2.387	Glucose, Mannose, Xylose, Galactose, Arabinose	Composed of β-glucans with β-(1→3) and β-(1→6) linkages	Cytotoxicity assays, flow cytometry, and immunofluorescence to assess cells activation and assessment of immune response markers in mouse blood and tissue	Macrophages and mouse	Inhibited NF-kB pathway, enhanced macrophage activity, and activated monocytes, enhancing phagocytosis and cytokine production	[59,79,85]
*Fucus* *vesiculosus*	Brown seaweed	735	Galactose, Glucose, Fucose, Xylose, Arabinose	Composed of α-(1→3) and α-(1→4) linkages	Cytokines assays, cell viability assay, Western blotting analysis, and RT-PCR	Macrophages, microglial cells, and Caco-2 cells	Reduced TNFα and IL-1B, activated dendritic cells, and activated monocytes	[60,87]
*Undaria* *pinnatifida*	Brown seaweed	97.9	Galactose, Fucose, Xylose, Mannose, Arabinose, Rhamnose	Composed of α-(1→3) and α-(1→4) linkages	Cartilage and bone destruction, tissue infiltration with inflammatory cells, and cytokines assay	T-cells, NK cells, mice, and breast cancer in rats	Inhibited NF-kB pathway, enhanced NK cell activity, and activated monocytes	[61,88,89]
*Chondrus* *crispus*	Red seaweed	0.12237	Galactose, Glucose, Xylose, Arabinose, Mannose	Composed of k- and γ-carrageenan	Enhancement of macrophage activity and ELISA	Macrophages and Caco-2 cells	Reduced TNFα and IL-1B and enhanced macrophage activity	[62,90,91,92]
*Flammulina* *velutipes*	Mushroom	7473.14	Fucose, Glucose, Mannose, Xylose, Arabinose	Composed of β-glucans	MTT assay, macrophage activity, cytokine assay, and antioxidant activity	Macrophages and mouse	Reduced TNFα and IL-6 and enhanced T-cells activity	[63,85]
*Sparassis* *crispa*	Mushroom	75	Galactose, Rhamnose, Mannose	Composed of β-glucans with β-(1→3) and β-(1→6) linkages	Mitochondrial function, macrophage activity, anti-inflammatory, and antioxidant activity	Macrophages and mouse	Inhibited NF-kB pathway and enhanced macrophage activity	[64,93]
** Cucurbita *moschata*	Pumpkin	18	Glucose, Galactose, Mannose, Arabinose	Composed of α-(1→4)- linked galacturonic acid residues	Intragastric injection of polysaccharide, 16S rRNA gene sequencing, and analysis of SCFA	Rats	Modulated gut microbiota and enhanced gut-associated lymphoid tissue	[65,82]
** Hordeum *vulgare*	Cereal grain	0.2120885	Glucose, Xylose, Arabinose	Composed of linear β-(1→3) and β-(1→4) linkages	Antioxidant activity, gut microbiota modulation, cytokine measurement, antibody production, and histopathological analysis	Macrophages and mouse	Reduced TNFα and IL-6, modulated gut microbiota, and activated monocytes via dectin-1 receptors	[66,94,95,96]
* Fragaria x *ananassa*	Strawberry	<50	Glucose, Galactose, Arabinose, Xylose, Rhamnose	Composed of backbone of α-(1→4) linked galacturonic acid residues	Pro-/anti-inflammatory cytokine levels secreted by LPS-stimulated macrophages cultured with SP and MP for 48 h were determined using ELISA method to evaluate anti-inflammatory effects of SP and MP. The Bcl-2/Bak (anti-/pro-apoptotic) protein levels in the cells were determined using Western blotting method to evaluate anti-apoptotic effects	Macrophages	Reduced TNFα and IL-1B and enhanced macrophage activity	[67,97]
* Morus alba	Mulberry	1.408–7.812	Glucose, Galactose, Arabinose, Xylose, Rhamnose, Mannose	Composed of various glycosidic linkages	Pro-/anti-inflammatory cytokine levels secreted by LPS-stimulated macrophages cultured with SP and MP for 48 h were determined using ELISA method to evaluate anti-inflammatory effects of SP and MP. The Bcl-2/Bak (anti-/pro-apoptotic) protein levels in the cells were determined using Western blotting method to evaluate anti-apoptotic effects	Macrophages	Reduced TNFα and IL-6 and enhanced macrophage activity	[68,97,98]
*Dendrobium* spp.	Orchid	136	Glucose, Galactose, Arabinose, Xylose, Rhamnose, Mannose	Composed of glucomannans with β-(1→4)-linked backbones	Cell culture-based assays to evaluate cell viability, anti-inflammatory effects, antioxidant activity, enzyme inhibition, pharmacokinetics, and toxicity assays	Macrophages and mouse	Inhibited NF-kB pathway, enhanced T-cell activity, and activated monocytes and cytokine production	[69,99,100]
*Amorphophallus konjac*	Konjac	≈1400–950	Glucose, Mannose	Composed of β-(1→4)-linked D-mannose and D-glucose units with acetyl groups	Analysis of NK cell lethality, cell proliferation, pinocytic activity of mouse macrophages, serum levels, cytokines assay, and protein levels assay	Macrophages and mouse	Reduced TNFα, IL-1B, and IL-6, inhibited NF-kB and MAPK pathway, and enhanced macrophage activity	[101,102,103]

* arabinose as a major compound; ** arabinose as a second major compound.
